# Mapping Historical Landslide Activity Using a Swin Transformer-Based Transfer Learning Approach

**DOI:** 10.3390/s26010293

**Published:** 2026-01-02

**Authors:** Fei Chen, Zhihua Liang, Zhengyuan Cheng, Hui Li, Cheng Zhong, Zekun Hu

**Affiliations:** 1 School of Surveying and Geoinformation Engineering, East China University of Technology, Nanchang 330013, China; 2 Jiangxi Provincial Natural Resources Surveying and Monitoring Institute, Nanchang 330002, China; 3 The School of Earth Sciences, China University of Geosciences, Wuhan 430074, China; 4 Badong National Observation and Research Station of Geohazards, China University of Geosciences, Wuhan 430074, China; 5 State Key Laboratory of Information Engineering in Surveying, Mapping and Remote Sensing, Wuhan University, Wuhan 430079, China

**Keywords:** historical landslide, transfer learning, swin transformer, cross domain, landslide inventory

## Abstract

Historical landslide inventory serves as a critical tool for analyzing landslide activity patterns and evaluating the long-term geological impacts of triggering events, including earthquakes, extreme weather events, and large-scale infrastructure projects. Although various methods—including visual interpretation, heuristic approaches, machine learning, and deep learning models—have been employed for landslide detection, efficient techniques for historical landslide mapping remain understudied. As a result, comprehensive historical landslide inventories continue to be scarce worldwide. In this study, we developed an advanced landslide detection model using a Swin Transformer architecture integrated with a Pyramid Segmentation Attention mechanism. Subsequently, we applied a network fine-tuning method to achieve cross-domain adaptation, enabling the reconstruction of a decadal-scale landslide inventory across the Wenchuan earthquake-affected region efficiently. Experimental results from the Wenchuan earthquake area demonstrate the proposed approach’s superior temporal transfer mapping performance compared to state-of-the-art models. The proposed historical map also exhibits high accuracy and completeness, offering significant value for analyzing landslide spatiotemporal activity and long-term regional stability. Findings reveal that landslides stabilized overall between 2008 and 2021, with key influences including altitude, slope, and aspect. The results lay the groundwork for regional stability analysis and eco-environment recovery, enabling informed decisions in urban planning and infrastructure investments.

## 1. Introduction

Landslides, triggered by earthquakes, heavy rainfall or anthropogenic activities, frequently cause substantial casualties, property damage, and infrastructure disruptions [1,2,3]. As the foundational step in landslide analysis [4,5], landslide mapping enables assessment of susceptibility, hazards, vulnerability and risks, making it critical for effective disaster mitigation [6]. Particularly, historical landslide inventory achieved through extended periods of systematic cataloging, yields insights into the historical distribution patterns, development trends, and influencing factors of landslides [7,8,9]. This enables accurate prediction and assessment of potential landslide occurrence locations, frequencies, as well as a deeper understanding of landscape evolution processes and mechanisms [10,11]. For regions affected by earthquakes or heavy precipitation, historical inventory is highly significant in assessing the long-term stability [12], which is crucial for making informed decisions regarding urban planning and infrastructure investments [13].

Traditional visual interpretation demonstrates operational feasibility and reliability, yet suffers from significant limitations including high subjectivity, low efficiency, and substantial costs [5,14]. Recent advances in machine learning have enhanced landslide identification efficiency by enabling automated extraction of multidimensional landslide features from remote sensing imagery, including spectral, textural, and morphological characteristics [15,16]. Widely adopted techniques include logistic regression, Bayesian approaches, support vector machines, and random forests [17]. Nevertheless, these models require extensive feature selection/optimization and parameter tuning, which constrains their application efficiency, scope, and reliability. Deep learning enhances intelligent landslide detection by hierarchically extracting image semantic features from low to high levels with minimal human intervention [18]. Object detection models such as the YOLO series identify landslides through bounding boxes and class labels, with accuracy enhanced by attention mechanisms and ensemble learning techniques [19]. Semantic segmentation architectures (e.g., SegFormer, DeepLabV3, U-Net) perform pixel-level classification of remote sensing imagery [20]. However, since supervised deep learning models require large volumes of high-quality labeled data, maintaining annotation consistency and accuracy under complex terrain and environmental conditions presents significant challenges [21]. To reduce sample size requirements, transfer learning—which leverages pre-existing knowledge to enhance performance in new domains—has been successfully employed in landslide mapping tasks [22,23,24,25].

Nevertheless, most landslide detection studies focus on single- or few-time landslide mapping, resulting in scarce historical landslide inventories [3]. Although some attempts at historical landslide mapping have been made, they often achieve neither high accuracy nor efficiency. For instance, an annual landslide detection approach has been employed to generate a historical landslide inventory for Taiwan [8]. Although this method ensures accuracy, it necessitates yearly sample collection and model retraining. Behling et al. [25] detected landslides by identifying dramatic changes in Normalized Difference Vegetation Index (NDVI) curve. Wang et al. [26] employed the Continuous Change Detection and Classification (CCDC) algorithm to develop a multi-year landslide inventory. Such regression-based analytical methods frequently struggle to accommodate complex interference from multiple factors—including cloud cover, atmospheric scattering, illumination conditions, and seasonal variations—due to their idealized assumptions about data patterns. These limitations often lead to noise and misclassification. Furthermore, pixel-level detection approaches lack holistic scene understanding, failing to correctly classify landslide surfaces with intact vegetation.

Given the widespread demand for historical landslide inventories, this study presents a practical integrated approach adapting a Swin Transformer architecture with a Pyramid Pooling Module (PPM), a Pyramid Segmentation Attention (PSA) mechanism, and full-scale skip connections for landslide detection. While PPM, PSA, and full-scale skip connections are individually mature techniques, their synergistic integration is targeted at addressing the unique challenges of long-term historical landslide mapping—specifically, weakened landslide spectral features, multi-scale variability of landslide bodies, and loss of low-dimensional details during cross-temporal transfer. This design differs from existing Transformer-decoder hybrid models (e.g., SegFormer, Mask2Former) in two key aspects: PSA simultaneously optimizes channel-wise and spatial attention for fused features from PPM and FPN, rather than relying on single-dimensional attention or simple concatenation; full-scale skip connections bridge encoder hierarchical outputs with decoder upsampled features via channel normalization, mitigating the loss of shallow terrain details critical for identifying long-term stable landslides. To enhance temporal generalization, we implement network fine-tuning for cross-temporal domain adaptation, transferring prior knowledge from a pre-trained model on source domain long-term landslide data. This strategy is compared with domain adaptation and self-supervised pre-training: domain adaptation focuses on spatial domain distribution shifts, less targeted at temporal feature drift of landslides; self-supervised pre-training requires massive unlabeled data, scarce for long-term multitemporal imagery in specific hazard regions. In contrast, network fine-tuning leverages labeled source data to learn generic landslide features and adapts via parameter optimization, balancing data efficiency and transfer performance. Finally, we validate the approach by generating a 2008–2018 landslide inventory and analyzing activity patterns. The results demonstrate stable performance across temporal scenarios, establishing this integrated method as a promising solution for large-scale historical landslide mapping.

## 2. Materials

### 2.1. The Study Area

The Wenchuan earthquake, measuring 7.9 in magnitude, struck on 12 May 2008, in the mountainous region of Sichuan Province, China. This seismic event stands as one of the most catastrophic earthquakes in recent Chinese history, causing extensive destruction and an immense human toll. Over 87,000 people were confirmed dead or missing, with millions more sustaining injuries. The earthquake occurred in a complex transition zone between the Tibetan Plateau (to the west) and the Sichuan Basin (to the east), where the Indian Plate collides with the Eurasian Plate, driving crustal shortening at ~4 mm/year. The Longmenshan Fault Zone (comprising the Wenchuan–Maoxian, Beichuan–Yingxiu, and Pingwu–Qingchuan faults) accommodated this compression through thrust and strike–slip mechanisms. The rupture propagated northeastward for ~90 s, with surface displacements exceeding 9 m vertically and 4.8 m horizontally. The steep topography (elevation gradients >4000 m over 50 km) and fractured bedrock exacerbated landslide susceptibility. Comprehensive insights into the tectonic setting, geological and geomorphological features of the area, and mechanisms of the Wenchuan earthquake are available in prior studies [27,28,29]. The earthquake triggered approximately 200,000 co-seismic landslides, spanning an area of 110,000 km^2^, and resulting in around 20,000 casualties [30].

**Figure 1:**
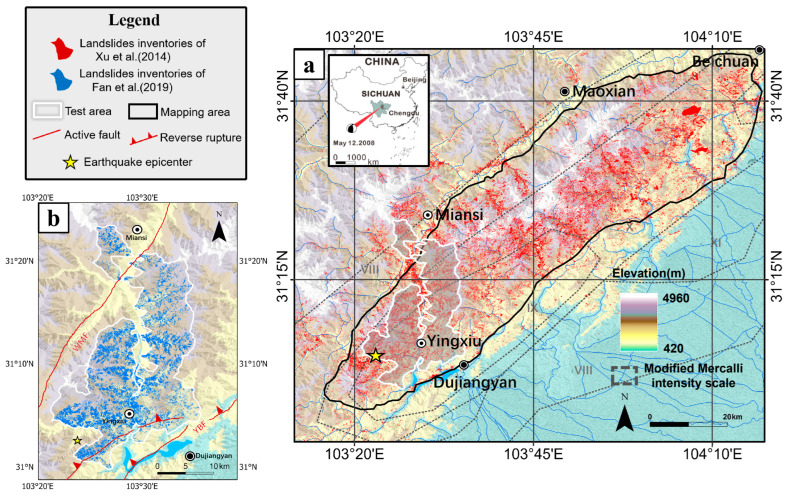
The study area: (**a**) the target domain and (**b**) the source domain [30,31]. The black line frame indicates the target domain, and the white line frame indicates the source domain. Active fault is marked with red solid lines, reverse rupture with orange dashed lines, earthquake epicenter with yellow star, and landslide inventories with different color shadings.

The target domain (Figure 1a), spanning from Yingxiu Town (the epicenter) to Beichuan County along the Longmenshan Fault, represents the most severely earthquake-affected area. Although covering only 3900 km^2^ (3.5% of the total earthquake-affected region), this area recorded 100,626 co-seismic landslides (51% of the total count), with a combined coverage of 663.95 km^2^ (58.6% of the total landslide area). Accurate mapping of historical landslides in this region is essential for evaluating the long-term seismic impacts and predicting future landslide occurrences, including their likely locations and timing. Such information is vital for evidence-based disaster risk reduction and strategic infrastructure planning.

The source domain comprises a smaller subregion of the earthquake-affected area (Figure 1b), where multitemporal landslide inventories produced by Fan et al. [31] are available. These inventories were compiled through visual interpretation of high-resolution imagery, including aerial photographs, SPOT, WorldView-2, Pléiades, and Landsat data spanning 2005, 2007, 2008, 2011, 2013, 2015, 2017, and 2018. We conducted additional manual interpretations for the years 2009, 2010, 2014, and 2016 using high-resolution Google Earth imagery [25], resulting in a continuous landslide inventory (Table 1).

### 2.2. Historical Samples Preparing

Sample data is indispensable for training and validating the performance of deep learning models. In the target domain, we initially obtained Landsat images captured during growing seasons from 2008 to 2018. Subsequently, we introduced histogram matching and color equalization techniques to alleviate color disparities resulting from seasonal variations and climate conditions between images acquired at different times. This approach effectively reduced the challenges associated with processing multi-year image data.

The reference sites for landslide sample collection, annotation, and model validation in this study prioritize regions with authoritative historical landslide inventories. Core reference site data is derived from Fan et al. [31], which achieves high annotation accuracy (exceeding 92%) through cross-validation of field surveys and remote sensing interpretation. To ensure data continuity and usability, reference sites must maintain multi-source remote sensing data availability (Landsat series, Google Earth) during the growing season (2008–2018), with cloud cover <10% and no severe atmospheric interference or data loss. This rigorous criterion guarantees clear delineation of landslide boundaries and spectral characteristics, providing high-quality data support for sample annotation and model training.

The irregular distribution of landslide inventory data within the study area presents significant challenges for sample generation. While traditional sliding window cropping methods can automatically produce large quantities of samples, they often generate numerous invalid samples containing null or spurious values. Simply applying a null-value ratio threshold to eliminate these problematic samples risks excessive false rejections, potentially discarding valuable training data and limiting the model’s ability to learn region-specific landslide characteristics. To address this issue, our study implements a novel strategy combining systematic grid-based image segmentation with manual sample screening. The grid-based approach first efficiently identifies areas with concentrated landslide occurrences, significantly reducing null and spurious values during the initial segmentation phase. Subsequent expert-led manual evaluation of each cropped sample ensures preservation of all geomorphologically meaningful data while avoiding the misjudgments inherent in automated filtering methods. This precision-curated dataset provides higher-quality training material for deep learning models, enabling more effective learning of regional landslide features and ultimately enhancing both model accuracy and generalization capability. Landslide sample images were cropped to 512 × 512 pixels, with approximately 170 samples generated per year.

Data augmentation refers to techniques that expand the training dataset by generating modified versions of existing samples, thereby increasing data diversity to mitigate overfitting and enhance model generalization. In deep learning, model performance is primarily evaluated by prediction accuracy on test datasets. Research demonstrates that feeding rotated or flipped versions of an image into learning algorithms yields distinct feature representations. Consequently, data augmentation provides additional training samples to prevent overfitting while improving generalization capability by exposing models to more varied data patterns. In this study, online data augmentation was employed to increase the diversity of training data and improve the model’s generalization capability. This augmentation includes the following: ➀ horizontal flipping, vertical flipping, diagonal flipping, or no flipping with a probability of 1/3; ➁ adjusting the brightness of the image by multiplying it by a random number ranging from 0.8 to 2.2 with a probability of 0.8; and ➂ calculating the average pixel value of the image after adjusting the brightness, then adjusting the image’s contrast with a probability of 0.8 by modifying contrast coefficients and average pixel values ranging from 0.8 to 2.2. In addition to the adopted online data augmentation techniques (flipping, brightness/contrast adjustment), the model training process shuffles the training set samples via random sampling in each epoch, ensuring no fixed pattern in the sample input order for each iteration.

### 2.3. Data Acquisition

Building on the study area definition (Section 2.1) and historical sample preparation requirements (Section 2.2), data acquisition is the foundational step for model training and historical landslide mapping. High-quality, consistent multitemporal remote sensing data is critical for capturing landslide spatiotemporal dynamics and supporting the transfer learning framework (Section 3.2), and this section details the data sources, time ranges, and extraction strategies for the source and target domains to align with the LandTrans model design (Section 3.1) and the study’s core goal of reconstructing a decadal-scale landslide inventory.

The source domain used multi-source high-resolution images—including aerial photographs, SPOT, WorldView-2, Pléiades, and Landsat series (Landsat 5/7/8 OLI/TM) data—to generate reliable labeled samples for pre-training. These datasets integrate multi-spectral and high spatial resolution features essential for distinguishing landslide boundaries from complex backgrounds and ensuring annotation accuracy. The source domain data spans 2005–2018 to support cross-temporal transfer learning: image data and landslide inventories for 2005, 2007, 2008, 2011, 2013, 2015, 2017, and 2018 were derived from Fan et al. [31], a validated study on Wenchuan earthquake-induced landslide mapping, while high-resolution Google Earth images were added for 2009, 2010, 2014, and 2016 to fill temporal gaps and ensure continuity of the time-series training dataset. Annotation of supplementary years followed Fan et al. [31] standards via manual visual interpretation, completed independently by two scholars.

The target domain focused on Landsat series images (Landsat 5 TM, Landsat 7 ETM+, Landsat 8 OLI) from 2008 to 2018, aligned with the study’s core focus on the 10-year post-Wenchuan earthquake landslide evolution cycle. The growing season was selected for stable vegetation coverage and low cloud cover, minimizing atmospheric/seasonal interference. Key data specifications include 30 m spatial resolution, multi-spectral bands for differentiating landslides from background features, and all image synthesis strictly followed the time range of the corresponding year, and only remote sensing data within a single year was used for processing without mixing cross-year spectral information, thus eliminating temporal lag issues.

To tackle multitemporal data variability and complex terrain, a hybrid extraction strategy (manual annotation + automatic segmentation) was adopted for accuracy and efficiency. The manual annotation phase (sample preparation) involved labeling landslide boundaries and attributes in the source domain (including supplementary years) via manual visual interpretation to ensure reliable training labels for supervised models like LandTrans. For automatic extraction (historical mapping), pixel-level semantic segmentation was applied to the target domain’s 2008–2018 Landsat images using the pre-trained LandTrans model for batch landslide extraction; preprocessing (histogram matching, color equalization) mitigated multitemporal spectral differences (consistent with Section 2.2 data augmentation), and postprocessing (small-area noise removal <10 pixels, morphological optimization) refined results into the final historical landslide inventory.

## 3. Methods

### 3.1. The LandTrans Model

**Figure 2:**
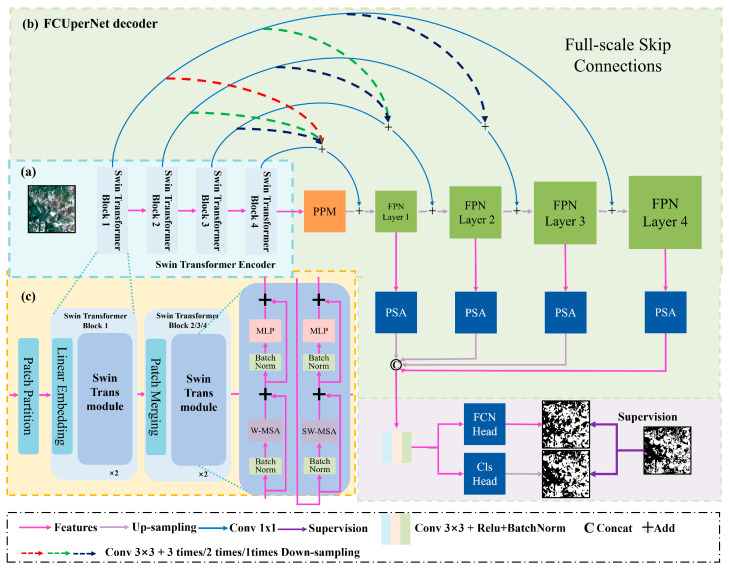
The structure of the proposed model. (**a**) Swin Transformer encoder. (**b**) the decoder. (**c**) the detail of Swin Transformer.

As illustrated in Figure 2, we employ a Swin Transformer backbone network to enhance the model’s capacity for capturing long-range dependencies. To overcome limitations of conventional feature pyramid decoders, we integrate a Pyramid Pooling Module (PPM) with an enhanced Feature Pyramid Network (FPN), connecting them to the encoder through full-scale skip connections. Additionally, we incorporate a Pyramid Segmentation Attention (PSA) mechanism for effective feature fusion. This architecture simultaneously addresses three key challenges: (1) detail loss from feature map downsampling, (2) low-dimensional feature information loss, and (3) semantic information degradation.

The Swin Transformer is a hierarchical vision transformer designed to address the limitations of traditional Vision Transformers (ViTs) and CNNs. It combines the global modeling capability of transformers with the locality and efficiency of convolutional operations, making it highly effective for tasks like image classification, object detection, and segmentation. As shown in Figure 2c, the Swin Transformer adopts a hierarchical construction method similar to convolutional neural networks (CNNs). It consists of four stages, each containing multiple Swin Transformer Blocks. At the start of each stage, the image resolution is reduced through a Patch Merging module. Within each Swin Transformer Block, window-based multi-head self-attention (W-MSA) and shifted window-based multi-head self-attention (SW-MSA) are alternately applied to compute features. The self-attention mechanism in Swin Transformer enables the model to consider all relevant elements—not just local or adjacent ones—when processing each element. This capability allows Swin Transformer to capture richer contextual information compared to traditional CNNs, leading to more precise modeling of global image features and long-range dependencies.

A Swin Transformer with four stages (comprising 2, 2, 6, and 2 Swin Transformer Blocks, respectively) is adopted as the backbone, initialized with weights pre-trained on ImageNet-1K to enhance feature extraction robustness. Each stage initiates with a Patch Merging module that halves the spatial resolution and doubles the channel count of the feature map, while W-MSA and SW-MSA are alternately applied within each Block with a fixed window size of 7, and the number of attention heads for stages 1 to 4 is configured as 3, 6, 12, and 24 respectively. Input images are converted into 16 × 16 pixel patches via the Linear Embedding layer with an embedding dimension of 96, and the channel dimension is progressively elevated to 192, 384, and 768 through subsequent Patch Merging operations in each stage.

The core formula of the SW-MSA mechanism is as follows:

(1) $$Attention\left(Q,K,V\right)=softmax(\frac{{QK}^{T}}{\sqrt{d_{k}}})\;V$$

Here, $Q\in R^{t_{q}\times d_{k}}$ is the query vector, $K\in R^{t_{k}\times d_{k}}$ is the key vector, and the term $\sqrt{d_{k}}$ serves as a scaling factor to prevent excessively large dot products that could lead to gradient vanishing.

The Patch Merging layer is responsible for downsampling the feature map by merging adjacent patches and increasing the number of channels. Assuming the input feature map has dimensions H × W × C:

(2) $$X_{merged}=Linear(Concat(X_{i},X_{i+1},X_{j},X_{j+1}))\;$$

Here, $X_{i},X_{i+1},X_{j},X_{j+1}$ are neighboring patches. They are concatenated and then linearly projected to generate a new feature representation.

The input feature map is divided into adjacent patches using a 2 × 2 window, and the 4 pixels within each window are concatenated along the channel dimension to form a feature matrix with dimensions H/2 × W/2 × 4C. A 1 × 1 convolution layer is then applied to compress the channel dimension to 2C, completing downsampling and channel enhancement. The weights of the convolution layer are randomly initialized, and the ReLU activation function is employed to introduce non-linearity into the feature transformation process.

**Figure 3:**
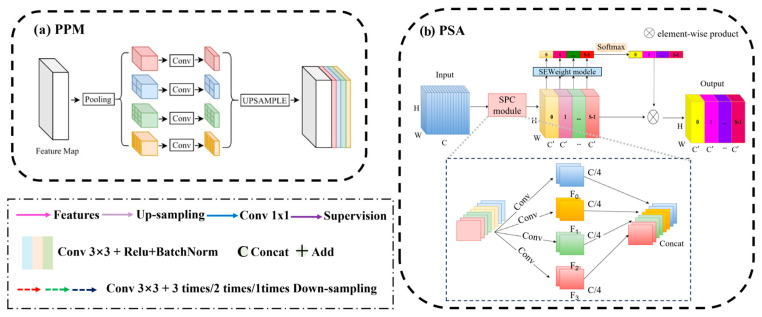
Details on PPM (**a**) and PSA (**b**).

As shown in Figure 3a, the PPM is designed to capture multi-scale contextual information from feature maps and enrich the representation of semantic features. The PPM operates by performing pooling operations at multiple spatial scales and then combining the resulting pooled features to create a richer representation. By pooling features at multiple scales, the PPM can capture both local and global contextual information, which is crucial for landslide detection where objects may vary significantly in size and context within an image. After pooling, the features from different scales are concatenated to form a unified representation that preserves both local and global context.

Given an input feature map $X\in R^{\left(H\times W\times C\right)}$, PPM performs multi-scale pooling, for each pyramid level n×n (e.g., 1×1,2×2,3×3,6×6):

(3) $$\begin{array}{c} P_{n}=AdaptiveAvgPool2d\left(X,output_{size}=\left(n,n\right)\right)\\ F_{n}={Conv}_{1\times 1}(P_{n})\\ U_{n}=Upsample(F_{n},size=(H,W)) \end{array}$$

Concatenate the original feature map with upsampled multi-scale features:

(4) $$X_{ppm}=Concat\left(X,U_{1},U_{2},U_{3},U_{4}\right)$$

FPN addresses the challenge of detecting objects at different scales within an image by combining features from different network layers to create a pyramid of feature maps with spatial resolutions at multiple scales. However, in the traditional FPN upsampling section, only high-level features are fused with preceding layer features through nearest-neighbor upsampling, which may result in loss of low-dimensional feature information. To address this issue, we use the full-stage long connection strategy like Unet3+, transforming the output feature maps of each layer of the backbone network into the upsampling layers of FPN through convolution and pooling and then adding them together (Figure 3b). This aids the model in acquiring more low-dimensional feature information, thereby enhancing segmentation accuracy. The FPN takes the outputs of the four stages of the Swin Transformer as inputs, corresponding to feature maps with resolutions of 1/4, 1/8, 1/16, and 1/32, respectively. The highest-resolution feature map (1/32) undergoes channel adjustment via a 1 × 1 convolution and is then upsampled to 1/16 resolution using bilinear interpolation. This upsampled feature map is added to the output feature map of the corresponding stage of the backbone network after unifying the channel dimension through another 1 × 1 convolution. This upsampling-fusion process is repeated to sequentially obtain fused feature maps with resolutions of 1/8 and 1/4. Each scale’s fused feature map is fed into an independent convolution layer to output prediction results, enabling joint decision-making based on multi-scale features.

PSA enhances feature representation in semantic segmentation by combining multi-scale spatial attention with channel-wise attention. It captures long-range dependencies while preserving fine-grained details, improving segmentation accuracy. In the model, the PSA module is used to reconstruct attention on channel and spatial dimensions of the outputs from the FPN and PPM). This attention reconstruction optimizes feature fusion, facilitating the effective integration of multidimensional and multi-scale contextual information, ultimately enhancing segmentation performance. In PSA, a Pyramid Pooling Module first aggregates features from different spatial scales to capture both local and global context information. These pooled features are then fed into a self-attention mechanism, which models the relationships between different spatial locations within the feature maps (Figure 3b).

### 3.2. Transfer Learning

Deep learning exhibits a strong reliance on large-scale training data, as substantial datasets are required to uncover underlying data patterns. The relationship between model scale and required data volume is nearly linear. In scenarios where training data is insufficient, transfer learning effectively addresses this challenge. Unlike conventional approaches, transfer learning neither requires training and test data to be independently and identically distributed, nor necessitates training the target domain model from scratch. This significantly reduces both data requirements and training time in the target domain. The typical workflow involves two key stages: First, a deep neural network model is trained on large-scale unlabeled data. Then, the pre-trained model is adapted to the new target task. Owing to the powerful feature representation capabilities of deep neural networks, this method automatically extracts latent patterns and features, enabling highly efficient knowledge transfer. Consequently, this approach not only achieves superior performance on new tasks but also dramatically reduces both training time and computational costs.

The transfer learning process consists of two stages: pre-training and fine-tuning. In the pre-training stage, the source domain’s 2005–2018 multitemporal labeled samples are used as inputs, with the AdamW optimizer configured with an initial learning rate of 0.0001 and weight decay of 0.05, a batch size of 16, and a total of 20,000 iterations. All network layers are updated during pre-training, with binary cross-entropy adopted as the loss function. Model performance is validated every 500 iterations using the validation set, and the model weights with the highest mIoU on the validation set are saved. In the fine-tuning stage, the pre-trained weights are loaded, and the first two stages of the Swin Transformer backbone are frozen to retain generic feature extraction capabilities, while the last two stages of the backbone, PPM, PSA, and FPN modules are fine-tuned for 40,000 iterations. The PolyLR learning rate scheduling strategy is employed, with an initial learning rate of 0.00001 that decays to 1 × 10^−6^ by the final iteration. Gradient clipping with a maximum norm of 0.01 is enabled in order to prevent gradient explosion, and transfer performance is evaluated every 5000 iterations using the target domain validation set (accounting for 20% of the target domain samples) by monitoring changes in mIoU and F1-score.

**Figure 4:**
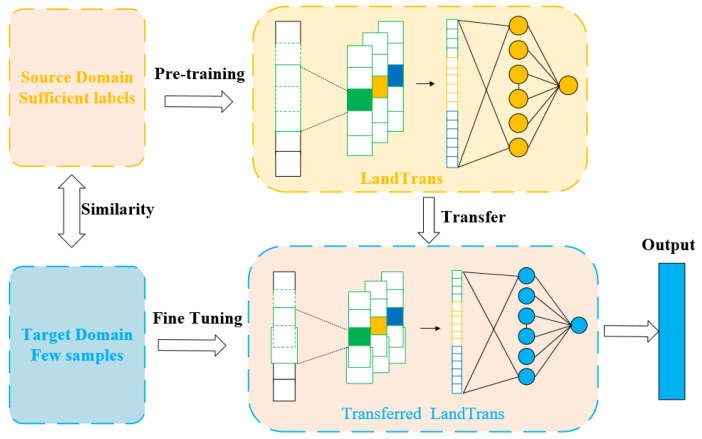
Network-based transfer learning.

Specifically, this study adopts network-based deep transfer learning (Figure 4). The methodology involves reusing components of a pre-trained network from the source domain—including its architecture and connection parameters—by integrating them into the target domain’s deep neural network as a functional submodule. The transferred subnetwork is subsequently updated via a fine-tuning strategy. This approach is grounded in the hypothesis that neural networks mimic the human brain’s processing mechanism through an iterative and continuous abstraction process. Early network layers act as feature extractors, with the extracted features exhibiting generic applicability.

### 3.3. Accuracy Assessment

This study employs several standard metrics to comprehensively evaluate landslide identification performance: Precision, Recall, mean Accuracy (mACC), Intersection over Union (IoU), mean IoU (mIoU), and F1 Score (see Table S1 for corresponding formulas). Precision measures the proportion of correctly detected positives among all predicted positives, reflecting the model’s overall reliability. Recall calculates what fraction of actual positives were correctly identified, which is particularly important when assessing costs associated with missed detections. mACC provides the average accuracy across all classes, offering a balanced view of model performance. IoU, a critical segmentation metric, quantifies the overlap between predicted and ground truth regions. mIoU, as the average IoU across classes, serves as one of the most important evaluation metrics for multi-class segmentation tasks. The F1 Score, as the harmonic mean of Precision and Recall, provides a comprehensive performance measure that balances both concerns.

These metrics are derived from pixel-level classification results using true positives (TPs), false positives (FPs), true negatives (TNs), and false negatives (FNs). Specifically: FP represents background pixels incorrectly classified as landslides, FN indicates landslide pixels misclassified as background, TP and TN denote correctly classified landslide and background pixels, respectively.

## 4. Experiment and Results

We conducted comparative tests on the LandTrans model against typical semantic segmentation models such as UNet, UNet3+, TransUNet, Pspnet, DeepLabv3+, Mask2Former, and Segformer to assess the performance of LandTrans. All models were trained and tested on a workstation equipped with two NVIDIA GTX3080super 8 GB GPUs, featuring cuDNN acceleration and NCCL distributed configuration. The models were developed and evaluated using the PyTorch 1.11.0 framework in Python 3.11, along with the MMsegmentation framework. This study does not adopt additional focal loss or weight balancing strategies, primarily because the ratio of landslide to non-landslide pixels in the samples is relatively balanced. The total area of the source domain in the study area is approximately 471 km^2^, with a cumulative landslide area of about 124.13 km^2^, resulting in an overall pixel ratio of approximately 1:2 between landslides and non-landslides in the samples, without extreme class imbalance. Therefore, the binary cross-entropy loss function can effectively characterize model prediction errors, and stable training can be achieved without additional balancing strategies. Due to the relatively balanced distribution of landslides and non-landslide within the test area, the training process utilized binary cross-entropy loss function and AdamW optimizer. The initial learning rate was set to 0.0001 with a weight decay of 0.05. Parameter-level learning rate adjustment was implemented, scaling the learning rate of the backbone network by a factor of 0.1. Additionally, gradient clipping was employed to prevent gradient explosion, with a maximum norm set to 0.01. PolyLR was adopted as the learning rate scheduling strategy, with the learning rate expected to decrease to 0.0001 over 40,000 iterations. This underscores the importance of gradually decreasing the learning rate for long-term training cycles.

### 4.1. Performance of the Proposed Model

Using models trained in the source domain in 2008, we conducted historical landslide mapping for the target domain from 2009 to 2018 and strictly adhered to the time independence principle for dividing the training and test sets, under which images from test years were completely excluded from the training set to avoid data overlap or temporal leakage. The corresponding accuracy results are presented in Table 2.

Table 2 indicates that the LandTrans model achieves the highest precision (74.33%), recall (80.18%), IoU (62.79%), mIoU (76.49%), and F-score (77.14%), demonstrating the best performance among all models. While Transformer-based models such as Mask2Former and SegFormer excel in capturing long-range dependencies, they may face challenges in obtaining adequate robust feature representations and modeling intricate relationships when operating with limited samples. This can result in decreased performance in historical landslide mapping tasks. In contrast, the hierarchical design of the Swin Transformer enables the capture of image features at various resolutions, thereby facilitating a more comprehensive understanding and representation of image content, particularly in scenarios with limited samples.

**Figure 5:**
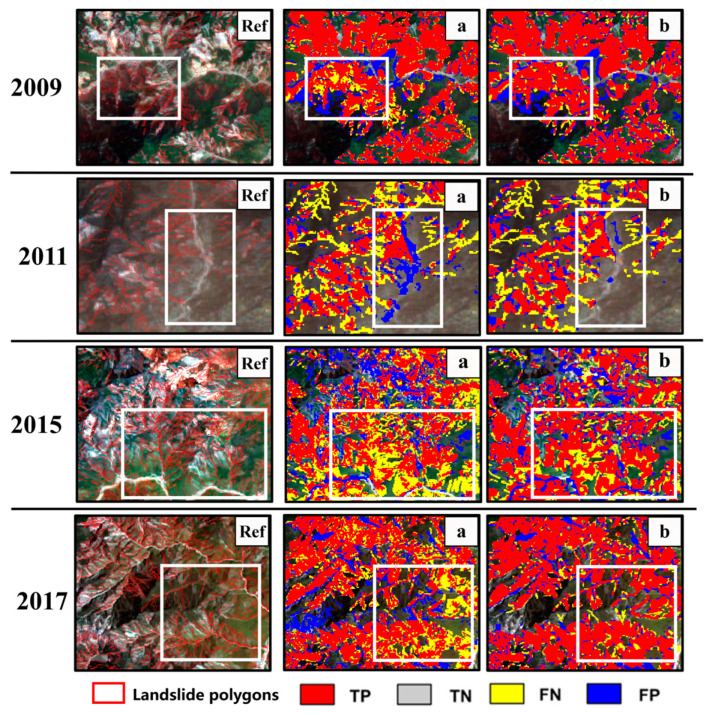
Historical landslide mapping samples. In this figure: “Ref” denotes validation samples, “a” shows UNet3+ results, “b” displays LandTrans results. To provide comprehensive results, we present different subregions from various years. The white frames in the figure denote highlighted regions focusing on key local details.

Table 3 demonstrates that UNet3+ maintains relatively stable performance during 2009–2011 (F1-score of 85.16% in 2009) but exhibits significant degradation after 2015 (F1-score dropping to 71.26% in 2015 and further to 67.65% in 2016). In contrast, LandTrans consistently retains robust performance across 2015–2018 (F1-score fluctuating narrowly between 71.98% and 79.77%). This performance divergence is intrinsically linked to the synergistic effects of spectral drift and vegetation recovery. First, the cumulative impact of spectral drift acts as a critical driver. Post-2015 climate shifts in precipitation patterns and temperature (Section 5.1) induced systematic spectral deviations in Landsat imagery. The CNN-based UNet3+, constrained by local receptive fields, struggles to adapt to long-term spectral heterogeneity. When test images diverge significantly from the training period (2008–2013), the model fails to align landslide spectral signatures, resulting in elevated false positives and missed detections. Conversely, LandTrans leverages the Swin Transformer’s hierarchical attention mechanism to capture long-range dependencies and mitigates spectral drift through histogram matching preprocessing (Section 2.2), thereby preserving stability. Second, vegetation recovery exacerbates performance decline. Accelerated ecological restoration after the Wenchuan earthquake led to dense vegetation coverage over 45.2% of pre-2015 landslides by 2015, masking landslide spectral signals with vegetation reflectance. UNet3+’s lack of multi-scale fusion and attention optimization mechanisms hinders discrimination between vegetated landslide remnants and normal vegetation. LandTrans, however, employs PPM multi-scale pooling and PSA channel–spatial dual attention to extract deep semantic features, compensating for vegetation-induced information loss and maintaining post-2015 detection accuracy. Additionally, thin cloud interference during 2013–2016 (Section 4.3) amplified UNet3+’s sensitivity to spectral noise, causing pronounced fluctuations in 2013 (74.45% F1-score) and 2016 (67.65% F1-score). Figure 5 further illustrates this disparity: UNet3+ produces significantly higher false positives and missed detections compared to LandTrans, which suppresses background noise while preserving precise boundary delineation. These findings underscore LandTrans’s superior capability in learning spatiotemporally complex landslide features and its resilience to spectral-ecological dynamics, offering a critical advancement for long-term landslide mapping.

### 4.2. Ablation Tests

We conducted ablation tests to comprehensively showcasing the necessity and value of our efforts. Specifically, we executed four comparative tests: S1, assessing the performance of with Swin transformer alone; S2, adding temporal samples processing (histogram matching and color equalization) to baseline; S3, adding PPM and PSA to baseline; and S4, a combination of S2 and S3. The existing ablation experiments (S1–S4) in this study are primarily aimed at verifying the synergistic improvement effect of “temporal sample preprocessing (histogram matching + color equalization)” and “PPM + PSA module fusion” on cross-temporal transfer performance, hence the experimental design focuses on the combined validation of temporal transfer-related improvements.

Compared to S1, after color equalization (S2), the mIOU metric increased from 75.27% to 75.61%, and mACC increased from 86.47% to 86.79% (Table 4). This indicates that color equalization effectively mitigated spectral differences between multitemporal images, thereby enhancing detection accuracy. After introducing the FCUperNet decoder (S3), compared to S1, there was a significant improvement in performance metrics. The mIoU increased from 75.27% to 76.15%, while mACC rose from 86.47% to 86.51%. Notably, Precision showed the most substantial enhancement, increasing from 72.43% to 74.68%. These results underscore the critical role of the FCUperNet decoder in augmenting spatial details and integrating features from multi-scale spatial channel attention. In S4, almost all metrics reached their highest levels compared to other tests. Particularly significant improvements were observed in mIoU and mACC. Compared to S1, the rises in all metrics were more pronounced, with mIoU increasing from 75.27% to 76.49%; and mACC increasing from 84.64% to 87.11%. This fully demonstrates the significance of both the color equalization and FCUperNet decoder in enhancing the model’s temporal transfer performance.

### 4.3. The Historical Landslide Inventory

**Figure 6:**
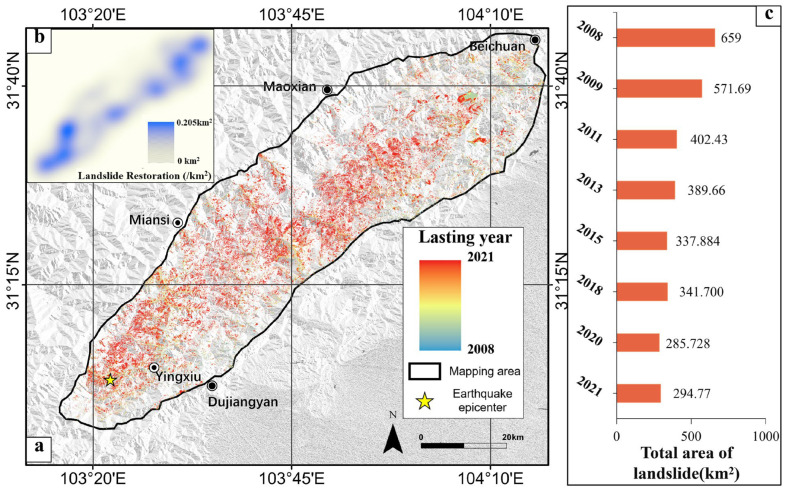
Historical landslide inventory for target domain, spanning from 2008 to 2018. (**a**) The lasting years of landslides. (**b**) The heatmap of landslide restoration. (**c**) The total area of landslides in different years.

Figure 6 presents the historical landslide inventory for the target domain, generated using the proposed model with transfer learning. This dataset represents a meticulously crafted product leveraging advanced deep learning technologies, offering significant value for analyzing the spatiotemporal activity of landslides in target domain. Additionally, it can serve as a valuable reference for conducting large-scale long-term map production, as well as for transfer learning studies and applications.

In 2008, a total of 100,626 co-seismic landslides were observed in TARGET DOMAIN, primarily concentrated along the Longmen Fault (as depicted in Figure 6a). While a significant number of landslides gradually diminished over time, larger landslides exhibited a tendency to endure for longer periods. Figure 6b illustrates that landslides restoration primarily occurred near the epicenter between 2008 and 2018, with minimal observation in the northeast region. This observation aligns with the distribution of co-seismic landslides, as the northeast region experienced fewer co-seismic landslides and, consequently, less restoration under similar conditions. Figure 6c demonstrates a rapid reduction in landslides total area of from 659 km^2^ in 2008 to 341.7 km^2^ in 2018. Linear trend analysis suggests an average annual restoration area of approximately 28 km^2^. We observed that the total landslide area did not decrease smoothly but exhibited multiple minor fluctuations. This is likely attributed to the possibility of some landslides reoccurring or new landslides occurring in the area due to various triggers such as precipitation, earthquakes, and human activities [32,33]. These factors may prolong the period of landslide activity in the study area. However, overall, after more than a decade, the active landslide area in the region has shown a steady declining trend [34,35,36]. By 2018, only 45.2% of the loose deposit caused by the earthquake still remained on slopes, signifying a substantial reduction in the earthquake’s impact on regional stability.

**Figure 7:**
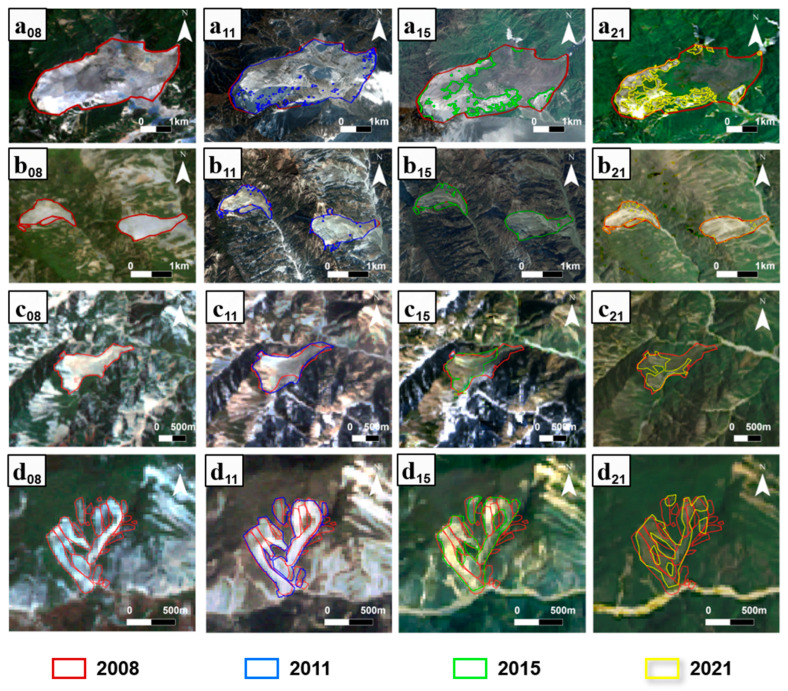
Samples of historical landslide inventory. (a–d) indicates different samples, while their subscript implies the year. For example, (a_11_) represents the region “a” in the year of 2011. Polygons in different colors represent landslides in different years.

Despite variations in hue and lighting conditions among images from various years, our method, aided by transfer learning, ensures accurate mapping for each year (Figure 7). Despite variations in the positions, shapes, and boundaries of landslides across different years, their distribution range or centroid has remained relatively stable since 2008, and the evolutionary footprint is very clear. These two aspects provide a solid foundation for accurately analyzing landslide activity or evolution trails over a long period. Figure 6 indicates all landslide areas have exhibited a decreasing trend from 2008 to 2018, which aligns with the overall evolutionary trend—a general slowdown in landslide activity—in the study area, as depicted in Figure 6. The quantitative area changes in typical landslide samples further confirm this trend, for sample a, the landslide area was 6.70 km^2^ in 2008, 6.45 km^2^ in 2011, 2.48 km^2^ in 2015, and 1.91 km^2^ in 2021; for sample b, the area was 1.10 km^2^ in 2008, 1.06 km^2^ in 2011, 1.04 km^2^ in 2015, and 1.05 km^2^ in 2021. for sample c, the area was 0.54 km^2^ in 2008, 0.70 km^2^ in 2011, 0.45 km^2^ in 2015, and 0.29 km^2^ in 2021.for sample d, the area was 0.47 km^2^ in 2008, 0.48 km^2^ in 2011, 0.41 km^2^ in 2015, and 0.3 km^2^ in 2021, directly supporting the conclusion that partial landslides weakened in activity during the study period. However, it is noteworthy that certain landslides continue to remain active or exhibit lower stability due to environmental factors or geological conditions.

## 5. Discussion

### 5.1. Historical Landslide Activity

Utilizing the LLM data, we conducted an analysis on the trends of landslide restoration and new occurrences (including re-occurrences) to evaluate regional landslide activity. In order to effectively showcase regional disparities, we implemented heatmaps to visually represent the spatial clustering degrees of these two events. This method aids in identifying hotspot areas or patterns by calculating the density of spatial points or events and illustrating it with colors of varying intensity and range. The spatial–temporal changes observed in the heatmaps of landslide restoration and new occurrences can broadly indicate the trend of landslide activity and contribute to understanding the evolution of regional stability.

**Figure 8:**
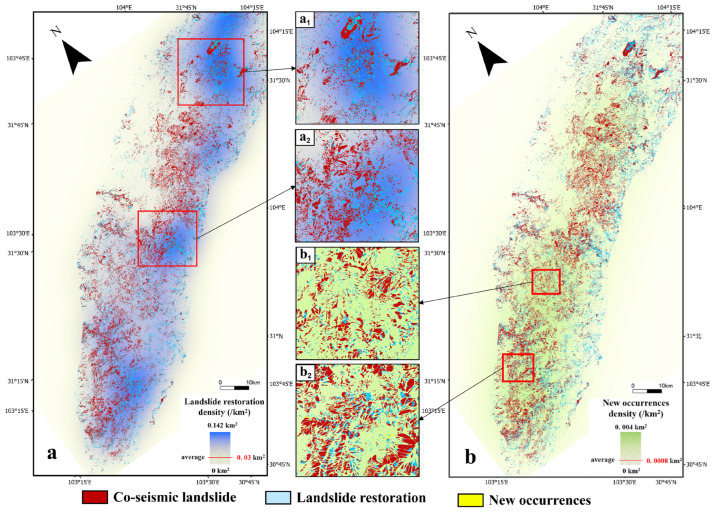
The heatmaps of landslide restoration (**a**) and new occurrences (**b**) spanning from 2008 to 2013. (**a_1_**) in (**a_2_**) represents two subregions of panel (**a**), while (**b_1_**) in (**b_2_**) indicates two subregions of panel (**b**). The values in the legend represent the annual density of changed landslides.

As depicted in Figure 8, the average density of landslide restoration from 2008 to 2013 reached 0.03 landslides/km^2^, while that of new occurrences was only 0.0008 landslides/km^2^. This indicates a notably higher rate of landslide restoration compared to new occurrences during this period. It suggests that following the earthquake, the majority of landslides tended towards restoration and ecological recovery, aligning with the changes in landslide area illustrated in Figure 6c. We observe that the distribution of hotspots for landslide restoration is extensive, spanning the southern epicenter, central, and northern regions, indicating a widespread trend. Although the distribution of hotspots for new occurrences is also wide, their numerical values are small, thus not indicating a significant trend in new landslides in the region.

As depicted in Figure S1, the average density of landslide restoration from 2013 to 2018 reached 0.0143, while that of new occurrences areas was higher than 0.0088. The disparity between them is not as pronounced as in the previous stage. However, the distribution of hotspots for landslide restoration remains more extensive compared to new occurrences. Both still contribute to a continuous decrease in the total landslide area, consistent with the changes in landslide area recorded in Figure 6c. During this period, the hotspot areas of landslide restoration are near the epicenter, while new occurrences are concentrated in the central region. The post-earthquake area has a large distribution of shallow deposits, which are highly susceptible to rainfall-induced landslides and debris flows [26,33,35,36,37]. New occurrences prolong the landslide activity cycle in the region, maintaining it in a state of instability for an extended period [32,33].

As illustrated in Supplementary Figure S2, the average density of landslide restoration from 2018 to 2021 reached 0.0132, whereas that of new occurrence areas exceeded 0.0083. We observed a decrease in the density of landslide restoration over time, primarily attributed to the gradual reduction in remaining landslides. In contrast, the density of new occurrences remained relatively stable, indicating the typical conditions of the area. Nonetheless, the total landslide area continued to decrease during this period. The map reveals that during these years landslide restoration predominantly occurred near the epicenter and in the central region, where new occurrences are more prevalent. On the map, the hotspot areas of new occurrences shifted from the central region to near the epicenter, potentially influenced by changes in precipitation distribution. In summary, the overarching trend of landslide activity since 2008 is clear: landslides have generally stabilized, with fluctuations attributed to heavy rainfall.

### 5.2. The Influences of Terrain Factors

**Figure 9:**
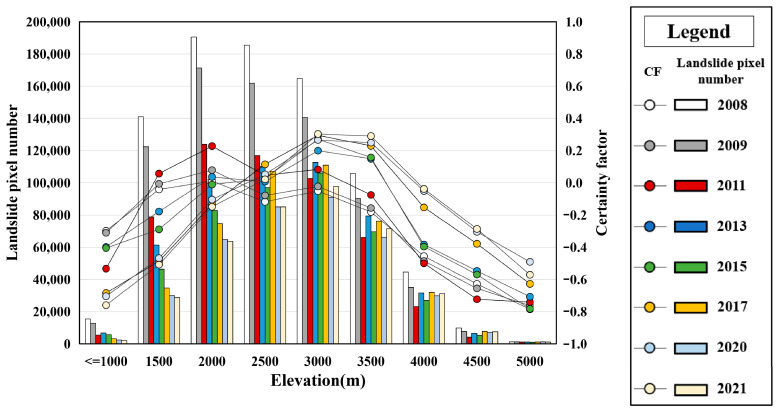
Relationship between landslide activity and elevation.

The spatial–temporal activity of landslides is influenced by natural conditions and human activities, with terrain factors playing a particularly prominent role [38,39,40,41]. This section examines three typical terrain factors—elevation, slope, and aspect—as examples to explore the impact of external factors on landslide activity in this area. The aim is to provide basic knowledge for the interested public and serve as a reference for further in-depth research. We utilize the Certainty Factor (CF) to quantitatively explore the relationship between landslide activity and controlling factors. The CF method, initially proposed by Shortliffe and Buchanan [42] and further improved by Heckerman [43], is a probabilistic function employed to assess the sensitivity of various factors influencing landslide occurrence. In this study, the CF value is calculated based on the number of landslides in each sub-class of each factor. The CF formula, modified by Lan et al. [44], is as follows (Equation (5)):

(5) $$CF=\frac{P_{a}-P_{s}}{P_{a}*\left(1-P_{s}\right)}\;\;\;\;\;\;\;P_{a}\geq P_{s}\;$$

(6) $$CF=\frac{P_{a}-P_{s}}{P_{s}*(1-P_{a})}{\;\;\;\;\;\;\;P}_{a}<P_{s\;}$$

where CF implies the probability of landslide activity, ranging from −1 to 1. A positive value indicates an increase in the certainty of landslide activity. Conversely, negative values correspond to a decrease in the certainty of landslide activity. A CF value close to 0 means that the prior probability is very similar to the conditional probability, making it difficult to provide any indication of the certainty of landslide activity. $P_{a}$ represents the active landslide area located within a specific sub-class (i.e., 30–40 degrees) of a terrain factor, divided by the total landslide area. $P_{s}$ represents the total area of active landslides divided by the study area’s total area. Figure 9, Figures S3 and S4 show the impact of different terrain factors on landslide activity from 2008 to 2021 using CF analysis.

Figure 9 indicates that seismic landslides in 2008 mainly occurred in mid- to high-altitude areas (1000 m to 3500 m). From 2008 to 2021, the area of active landslides decreased significantly below 3000 m, but there was little change above 3500 m. Over time, most landslide areas gradually disappeared, but landslide activity maintains significance in high-altitude areas. The CF values for landslides in 2008 began to decline once the elevation exceeded 2000 m. However, the turning point for CF values in subsequent years gradually increased. For example, CF values in 2013 and 2015 began to decrease at elevations exceeding 3000 m, while CF values after 2017 decreased only when elevations surpassed 3500 m. This pattern is attributed to the environmental conditions of high-altitude areas, characterized by low temperatures, sparse precipitation, and slow vegetation growth. These factors hinder the natural restoration of landslides, thereby maintaining them in an active state.

As depicted in Figure S3, landslides were predominantly concentrated within the slope range of 20° to 50° throughout all observed years. Prior to 2015, the majority of active landslides occurred within the slope range of 30° to 40°, whereas post-2015, they were primarily situated within the 40° to 50° range. This shift suggests that landslides occurring on gentler slopes experience more rapid restoration, as these slopes are better equipped to retain soil and moisture, facilitating improved vegetation recovery. In contrast, steep slopes present obstacles to vegetation recovery, resulting in landslides often leaving exposed deposits for prolonged periods, consequently heightening susceptibility to subsequent sliding events. Higher CF values indicate a greater likelihood of landslide occurrence. Figure S3 illustrates that steep slopes tend to exhibit larger CF values, whereas gentle slopes typically have smaller ones. This discrepancy arises from the greater stability of deposits on gentle slopes, which facilitates ecological restoration and reduces the likelihood of reactivation. In contrast, steep slopes encounter greater challenges in sustaining stability and ecological restoration, leading to increased landslide activity.

Figure S4 illustrates that the eastern and southern slopes exhibit higher landslide areas and CF values compared to the northern and western slopes, where the values are notably lower. Nonetheless, there has been a significant decrease in the area of active landslides in each direction from 2008 to 2021. The CF values of post-earthquake landslides diminish more rapidly on the northern and western aspects, suggesting a lower likelihood of continued activity on these slopes. This distribution and activity of landslides across different directions may be attributed to factors such as directional peak ground acceleration (PGA) and the direction of seismic fault slip [27,30]. The concentration of co-seismic landslides tends to rise with increasing horizontal PGA values, where ground motion with larger horizontal components may result in extensive damage on slopes [45,46]. PGA values vary across different directions; for instance, at the Wolong station located 18 km northwest of the epicenter, the maximum accelerations recorded were 957.7 Gal, 652.9 Gal, and 948.1 Gal for the EW, NS, and UD directions, respectively [47]. Additionally, disparities in sunlight exposure, evaporation rates, precipitation patterns, and discontinuities in various directions also influence landslide restoration processes [48].

### 5.3. Limitations and Future Works

This study has several inherent limitations. First, the research primarily focused on the area affected by the Wenchuan earthquake, without considering other seismic regions. Given the differences in seismic characteristics, climate types, vegetation coverage, or altitude gradients across various regions, the performance of the LandTrans model may decline in other areas. Future research will incorporate multi-regional samples to expand the training dataset and enhance the generalization capability of the LandTrans model.

Second, the 30 m spatial resolution of Landsat data results in lower detection capability for small-scale landslides (<2000 m^2^). Although these small landslides account for a relatively small proportion of the total area and have limited impact on overall trend analysis, they still affect the comprehensiveness and accuracy of the historical landslide inventory. In response, future studies will consider integrating high-resolution data (e.g., Sentinel-2) to improve the detection capability for small landslides.

Third, the current analysis of landslide surface ecological recovery primarily focuses on topographic factors (elevation, slope, aspect), while long-term driving factors such as land use changes and global climate change have not been fully incorporated, limiting the depth of interpretation of landslide evolution mechanisms. Future research needs to introduce more dimensions of influencing factors (particularly climatic factors such as rainfall and temperature) to strengthen the mechanistic analysis of landslide surface ecological recovery.

Fourth, although this study compared the performance differences among various models, it did not quantitatively evaluate factors affecting model performance, such as label bias, atmospheric effects, and vegetation changes. Detailed analysis of confidence maps or error distribution maps under different influencing factors is also lacking. Future research will conduct dedicated in-depth analysis to specifically examine the mechanisms by which external factors influence model uncertainty.

## 6. Conclusions

Mapping tens of thousands of historical landslides in a major earthquake-affected region presents a significant challenge. In this study, we developed LandTrans, an integrated approach adapting the Pyramid Pooling Module (PPM) and Pyramid Segmentation Attention (PSA) mechanism for targeted multi-scale feature fusion in long-term landslide mapping. Leveraging network-based deep transfer learning, we efficiently generated a comprehensive historical landslide inventory (2008–2021) for the target domain. Our experimental results demonstrate three key findings: LandTrans achieves reliable landslide identification and maintains stable performance over extended time periods, benefiting from the synergistic integration of mature components tailored to address temporal feature variability in historical mapping tasks. Comparative verification and validation confirm that the proposed method delivers high-quality, complete results, providing substantial value for spatiotemporal landslide activity analysis and long-term regional stability assessment. The results reveal that landslides have generally stabilized from 2008 to 2021, with fluctuations attributed to heavy rainfall, and the restoration process is influenced by altitude, slope, and aspect.

## Figures and Tables

**Table 1:** The historical inventory of source domain.

Year	2005	2007	2008	2009	2010	2011	2013	2014	2015	2016	2017	2018
Number	132	71	8924	8034	7617	9753	10,029	9644	10,124	9736	10,128	10,136
Area (km^2^)	0.72	1.02	124.13	117.2	113.02	88.11	110.79	118.58	125.43	125.03	125.08	123.85

**Table 2:** The average accuracy of transfer learning from 2009 to 2018.

Method	Precision (%)	Recall (%)	mACC (%)	IoU (%)	mIou (%)	F1-Score (%)
Mask2Former	64.43	71.05	81.03	51.04	68.61	67.58
PSPNet	69.26	69.87	81.6	53.33	70.49	69.56
Unet	68.33	74.26	83.42	55.24	71.45	71.17
TransUNet	68.95	73.21	83.06	55.06	71.49	71.02
SegFormer	69.44	73.44	83.24	55.51	71.76	71.39
DeepLabV3+	69.63	73.20	83.68	55.69	71.78	71.06
UNet3+	71.47	75.08	84.64	58.19	73.51	73.57
**LandTrans** (Ours)	**74.33**	**80.18**	**87.11**	**62.79**	**76.49**	**77.14**

Bold text indicates the optimal performance.

**Table 3:** The performance of models in specific years.

Year	LandTrans						UNet3+					
	Pre. (%)	Rec. (%)	mA. (%)	IoU (%)	mI. (%)	F1. (%)	Pre. (%)	Rec. (%)	mA. (%)	IoU (%)	mI. (%)	F1. (%)
**2009**	80.18	87.99	91.68	72.27	82.36	83.90	**81.39**	**89.31**	**92.48**	**74.16**	**83.84**	**85.16**
**2010**	**74.88**	**83.83**	**89.13**	**65.43**	**78.46**	**79.10**	73.67	83.35	88.72	64.22	77.66	78.21
**2011**	**67.21**	**77.91**	85.30	**56.45**	**72.69**	**72.17**	66.46	78.58	85.48	56.27	72.50	72.02
**2013**	**74.72**	**80.85**	**87.41**	**63.48**	**76.82**	**77.66**	72.50	76.50	85.05	59.30	74.15	74.45
**2014**	**68.31**	**81.08**	**86.84**	**58.92**	**74.00**	**74.15**	66.46	77.16	84.75	55.53	71.95	71.41
**2015**	**76.05**	**76.00**	**85.15**	**61.33**	**75.26**	**76.03**	71.81	70.72	82.05	55.35	71.32	71.26
**2016**	**68.91**	**75.33**	**84.14**	**56.22**	**72.32**	**71.98**	64.60	71.01	81.47	51.12	68.91	67.65
**2017**	**79.55**	**79.99**	**87.55**	**66.34**	**78.57**	**79.77**	75.97	73.83	84.14	59.85	74.39	74.88
**2018**	**78.90**	**79.12**	**87.06**	**65.30**	**77.92**	**79.01**	75.55	72.98	83.71	59.04	73.90	74.24

Bold text indicates the optimal performance.

**Table 4:** Ablation experiment results *.

Test	Precision (%)	Recall (%)	mACC (%)	IoU (%)	mIoU (%)	F1 Score (%)
S1	72.43	79.45	86.47	61.00	75.27	75.78
S2	72.70	80.05	86.79	61.54	75.61	76.19
S3	74.68	78.76	86.51	62.16	76.15	76.67
S4	**75.33**	**80.18**	**87.11**	**62.79**	**76.49**	**77.14**

* The data in the table represent the average accuracy metrics for testing years (2009–2018). Bold text indicates the optimal performance.

## Data Availability

The historical landslide inventory maps for the Wenchuan earthquake-affected region are publicly available through Figshare (https://doi.org/10.6084/m9.figshare.25579827.v1). The deep learning models were implemented using PyTorch within a Python environment. The complete source code for the proposed methodology has been deposited in Figshare (DOI: https://doi.org/10.6084/m9.figshare.25579818.v1).

## Supplementary Materials

The following supporting information can be downloaded at https://www.mdpi.com/article/10.3390/s26010293/s1, Figure S1: Heatmaps of landslide restoration (a) and new landslide occurrences (b) during 2013–2018; Figure S2: Heatmaps of landslide restoration (a) and new landslide occurrences (b) during 2018–2021; Figure S3: Relationship between landslide activity and slope gradient; Figure S4: Relationship between landslide activity and aspect; Table S1: Formulas for accuracy assessment indicators of landslide mapping.
