# Supplementary material for: Mapping Historical Landslide Activity Using a Swin Transformer-Based Transfer Learning Approach
Source: Sensors (Basel). 2026 Jan 2;26(1):293. doi: 10.3390/s26010293 (PMC12788299; doi:10.3390/s26010293)
Supplement: Supplementary file 1 [file sensors-26-00293-s001.zip › sensors-4038960-supplementary.pdf]

SUPPLEMENTARY INFORMATION

"Mapping Historical Landslide Activity Using a Swin Transformer-Based Transfer Learning Approach"

This file includes:

Figures S1-S4.

Table S1.

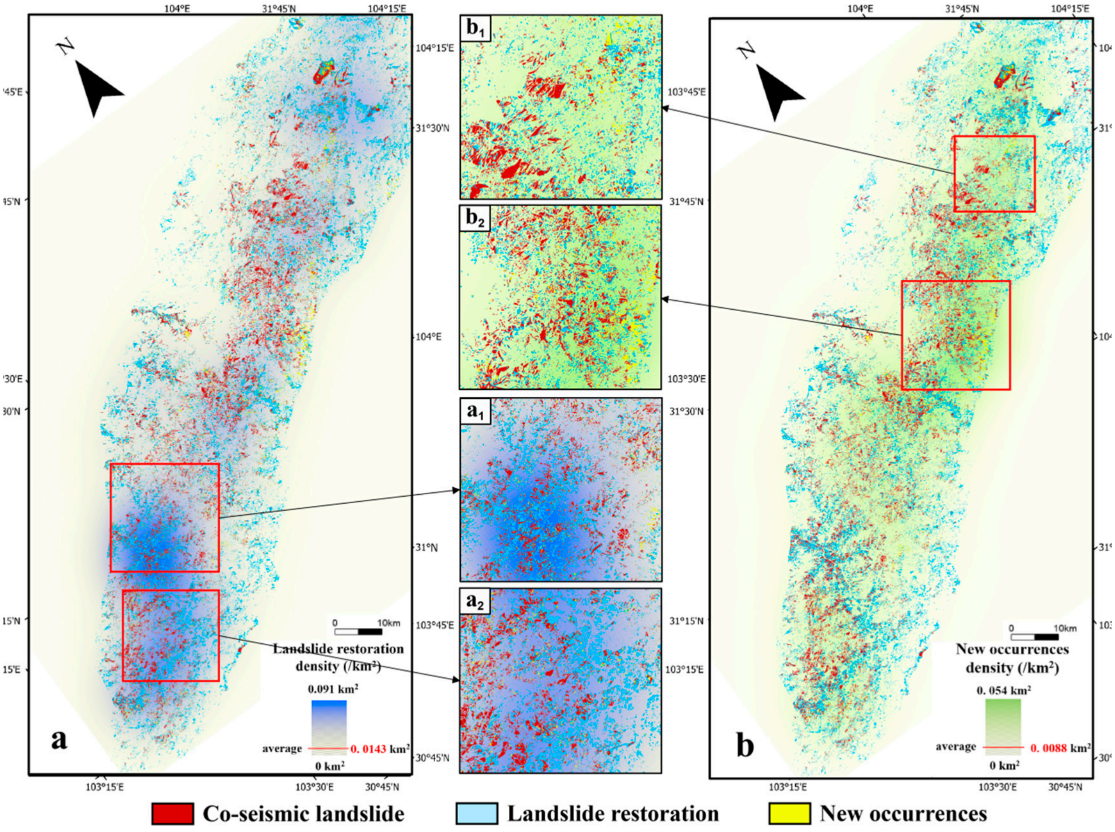

Figure S1. the heatmaps of landslide restoration (a) and new occurrences (b) spanning from 2013 to 2018.

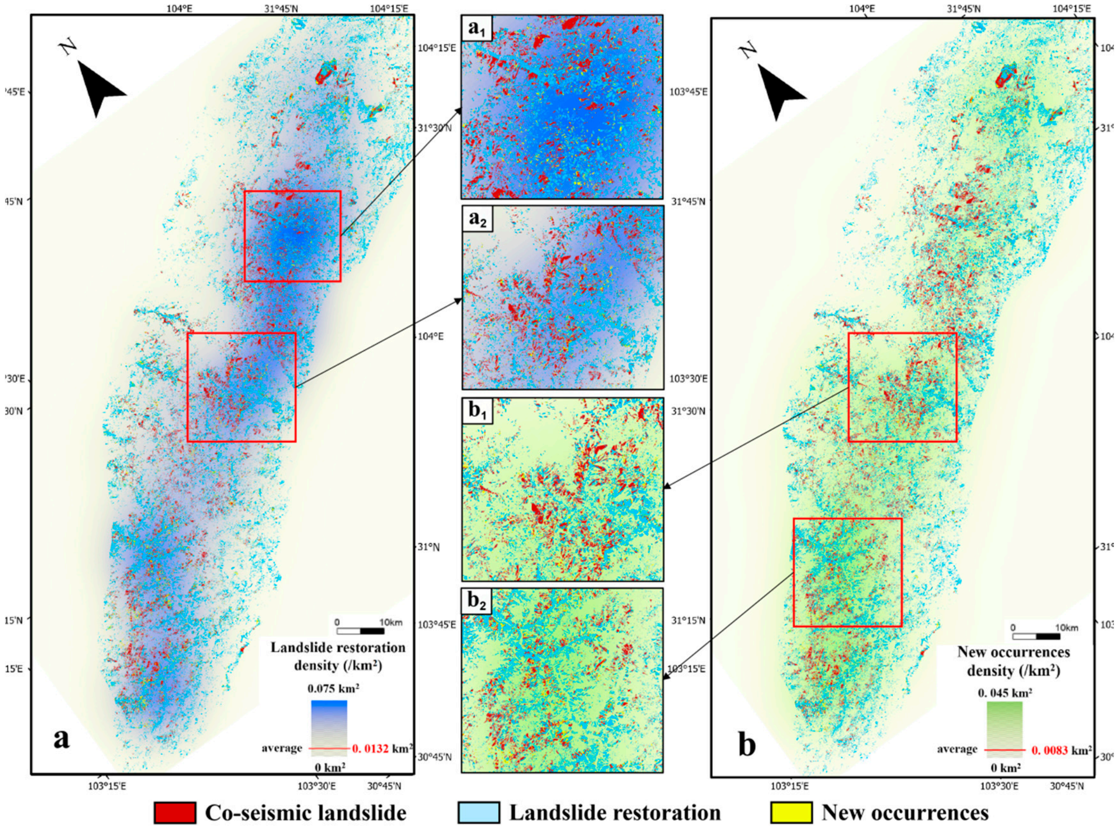

Figure S2. the heatmaps of landslide restoration (a) and new occurrences (b) spanning from 2018 to 2021.

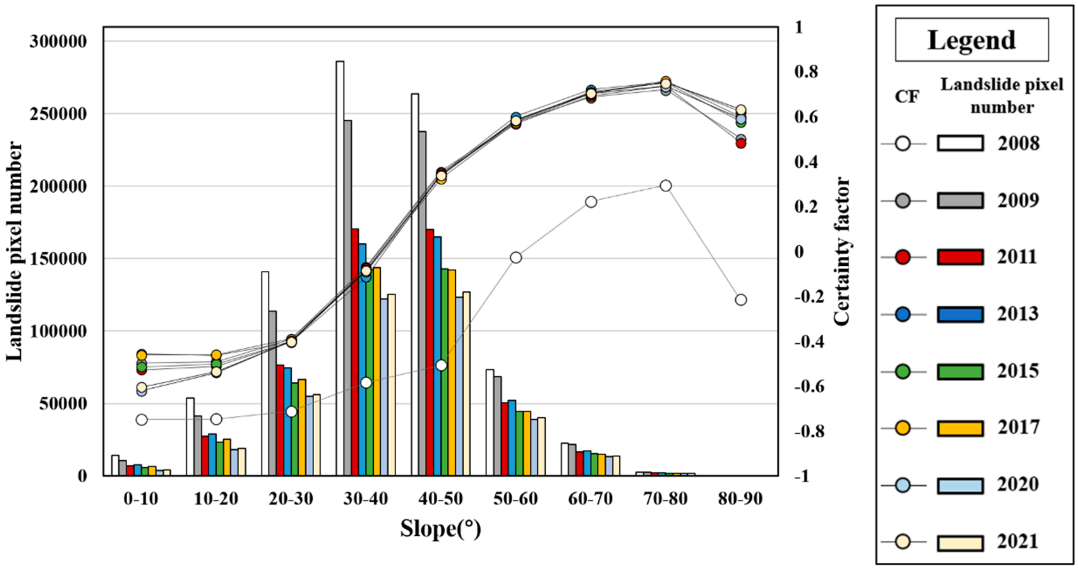

Figure S3. Relationship between landslide activity and slope.

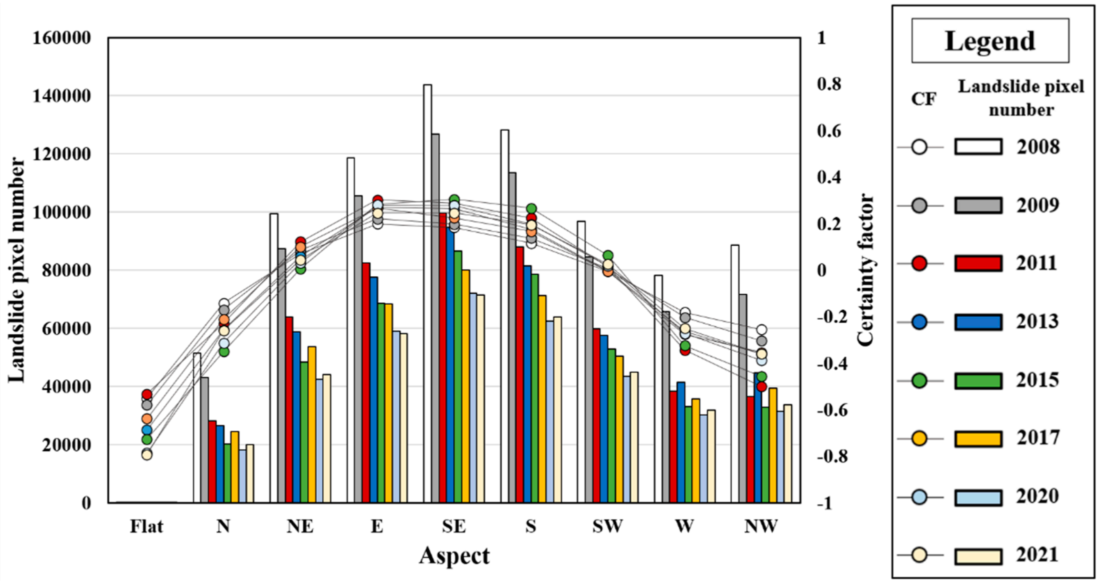

Figure S4. Relationship between landslide activity and aspect

Table S1. Formulas for accuracy assessment indicators

Indicators	Formulas
$mACC$	$\frac{1}{N}\sum_{i=1}^{N}\frac{TP_i + TN_i}{TP_i + TN_i + FP_i + FN_i}$
$Precision$	$TP/(TP + FP)$
$Recall$	$TP/(TP + FN)$
$F1Score$	$\frac{2\ \cdot Precison \cdot Recall}{Precison + Recall}$
IoU	$\frac{TP}{TP + FP + FN}$
$mIoU$	$\frac{1}{N}\sum_{i=1}^{N} IoU_i$
